# Twist-to-Bend Ratios and Safety Factors of Petioles Having Various Geometries, Sizes and Shapes

**DOI:** 10.3389/fpls.2021.765605

**Published:** 2021-11-11

**Authors:** Max Langer, Mark C. Kelbel, Thomas Speck, Claas Müller, Olga Speck

**Affiliations:** ^1^Plant Biomechanics Group @ Botanic Garden, University of Freiburg, Freiburg, Germany; ^2^Cluster of Excellence livMatS @ FIT – Freiburg Center for Interactive Materials and Bioinspired Technologies, University of Freiburg, Freiburg, Germany; ^3^Department of Microsystems Engineering – IMTEK, University of Freiburg, Freiburg, Germany

**Keywords:** twist-to-bend ratio, safety factor, petiole, biomechanics, body plan, geometry, size, shape

## Abstract

From a mechanical viewpoint, petioles of foliage leaves are subject to contradictory mechanical requirements. High flexural rigidity guarantees support of the lamina and low torsional rigidity ensures streamlining of the leaves in wind. This mechanical trade-off between flexural and torsional rigidity is described by the twist-to-bend ratio. The safety factor describes the maximum load capacity. We selected four herbaceous species with different body plans (monocotyledonous, dicotyledonous) and spatial configurations of petiole and lamina (2-dimensional, 3-dimensional) and carried out morphological-anatomical studies, two-point bending tests and torsional tests on the petioles to analyze the influence of geometry, size and shape on their twist-to-bend ratio and safety factor. The monocotyledons studied had significantly higher twist-to-bend ratios (23.7 and 39.2) than the dicotyledons (11.5 and 13.3). High twist-to-bend ratios can be geometry-based, which is true for the U-profile of *Hosta* x *tardiana* with a ratio of axial second moment of area to torsion constant of over 1.0. High twist-to-bend ratios can also be material-based, as found for the petioles of *Caladium bicolor* with a ratio of bending elastic modulus and torsional modulus of 64. The safety factors range between 1.7 and 2.9, meaning that each petiole can support about double to triple the leaf’s weight.

## Introduction

The petioles of foliage leaves fulfill various functions with sometimes contradictory demands. Their functions include aligning the lamina to the sun for photosynthesis (Niinemets and Fleck, 2002), vascularly connecting the lamina to the stem, supporting the weight of the lamina and elastically yielding under wind load to prevent the leaf from tearing. Of particular importance is the mechanical compromise of high flexural rigidity, which allows the petiole to be stiff enough under the bending load of the self-weight of the leaf (Vogel, 1992; Niklas, 1999), and a comparatively low torsional rigidity, which enables the petiole to be flexible enough to avoid damaging influences by wind loads (Vogel, 1989; Niklas, 1996). This biomechanical trade-off is reflected in the dimensionless twist-to-bend ratio (*EI/GJ*) (Vogel, 1992), which describes the flexural (bending) rigidity (*EI*) compared with the torsional rigidity (*GJ*) (Wainwright et al., 1976; Vogel, 1992; Etnier, 2003).

Another important mechanical aspect of biological structures is the dimensionless safety factor (*SF*), which estimates the maximum carrying capacity of the structures. In other words: the safety factor describes the extent to which the structure can carry more than its own static load (Gere, 2004). Both the twist-to-bend ratio *EI/GJ* and the safety factor *SF* are dimensionless variables and therefore allow comparisons to be made between various structures (Vogel, 1992, 1995, 2007; Niklas, 1999; Etnier, 2003). This is crucial as leaves and their petioles show a wide variety of morphological and anatomical characteristics. One aspect is the spatial configuration of the lamina and petiole, described by Langer et al. (2021) as three-dimensional (3D) for peltate leaves and two-dimensional (2D) for leaves in which the petiole is attached to the lamina base. Moreover, marked differences are found between the cross-sectional geometries of petioles, which can be circular, elliptical or U-profiled (Vogel, 1992; Ennos et al., 2000; Pasini and Mirjalili, 2006). Finally, differences occur in the body plan of herbaceous plants.

The term “body plan” was coined by Drost et al. (2017) and describes morphological features shared between species within a phylum. In this study, it is used to describe the internal arrangement of the vascular tissues in relation to mono- and dicotyledonous species.

The aspects mentioned above in turn influence the mechanics of the petioles. The configuration affects the way in which the applied loads act on the petioles, whereas the geometry, size and shape of the petioles affect their geometric characteristics, such as the axial (*I*) and polar (*J*) second moments of area and the torsion constant (*K*). The torsion constant (*K*) comes into play because, unlike the polar second moment of area (*J*), it takes into account the warping of non-circular structures under torsional loading and thus is often much smaller than *J* (Young et al., 2002). The body plan includes the arrangement of the vascular bundles that represent a type of strengthening tissue, which influences the material properties of the leaf, such as the bending elastic modulus (*E*) and torsional modulus (*G*).

We aimed to answer to the following scientific question: “How do cross-sectional geometry, sizes and shapes of petioles influence their twist-to-bend ratio and safety factor?”. Therefore, we selected four distinct types of petioles having a mono- or dicotyledonous body plan and/or a 3-dimenisonal (=peltate) or 2-dimensional (=petiole attached at the basis of the lamina) configuration of petiole and lamina. We obtained the necessary data through morphological and anatomical investigations and by two-point bending tests and torsional tests. In addition to the previously existing formula for the safety factor of vertically oriented structures, we provide, for the first time, an equation to calculate the safety factor of naturally horizontally oriented petioles. For the calculation of the twist-to-bend ratio, we did not use the polar second moment of area, as is usually the case, but the torsion constant, as the latter takes into account the warping of the structure during torsion.

## Materials and Methods

### Plant Material

**FIGURE 1:**
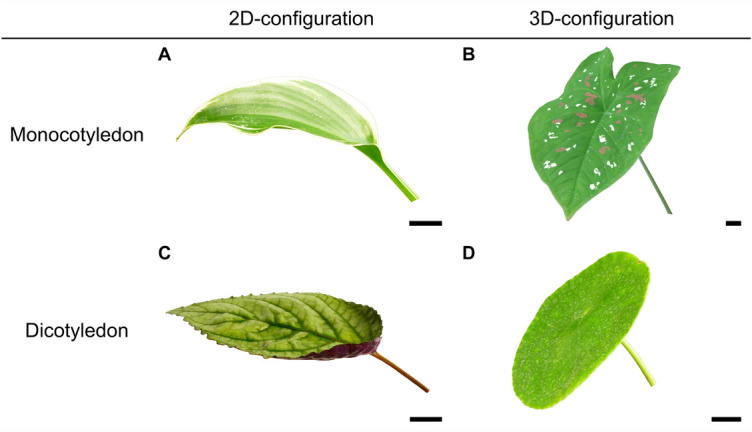
Leaf morphology of **(A)** *Hosta* × *tardiana* ‘El Niño’, **(B)** *Caladium bicolor*, **(C)** *Hemigraphis alternata*, and **(D)** *Pilea peperomioides*. Scale bar is 20 mm.

Plants of the species *Hosta* x *tardiana* ‘El Niño’ Piet Warmerdam (patent PP14632) (hereafter *H. tardiana*), *Caladium bicolor* Vent. (hereafter *C. bicolor*), *Hemigraphis alternata* (L.) Hallier f. (hereafter *H. alternata*), and *Pilea peperomioides* Diels (hereafter *P. peperomioides*) were cultivated in the greenhouse of the Botanic Garden (University of Freiburg, Germany). These four species were selected based on the same criteria as those described by Langer et al. (2021): (1) two species of each body plan (monocotyledonous and dicotyledonous) having a foliage leaf with either a 2D or 3D spatial configuration of petiole and lamina, (3) herbaceous, (4) perennial, and (5) easy to cultivate to provide sufficient material for experimentation. One random leaf of each of the 25 plants studied per species was investigated (Figure 1).

### Morphology

We weighed the lamina and petioles on a digital balance (accuracy ± 0.001 g). The weight ratio *WR* of the lamina *w*_*lamina*_ to the petiole *w*_*petiole*_ was calculated according to:


(1) $$WR=\frac{w_{lamina}}{w_{petiole}}$$

The diameters in the lateral direction *d*_*lateral*_ and in the adaxial-abaxial direction *d*_*adaxial*_ of each leaf stalk (petiole) were measured every 0.5 cm for *H. alternata*, every 1 cm for *H. tardiana* and *P. peperomioides* and every 3 cm for *C. bicolor* by a digital caliper (accuracy ± 0.01 mm). The aspect ratio *AR* of these perpendicular diameters gives an indication of the cross-sectional shape of the petioles, i.e., an *AR* of 1 means that the cross-section is as wide as it is high. This ratio was calculated as follows:


(2) $$AR=\frac{d_{lateral}}{d_{adaxial}}$$

**FIGURE 2:**
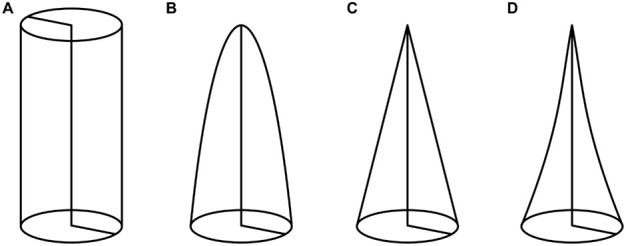
Tapering modes α of various slender structures: **(A)** α = 0 represents a circular cylinder, **(B)** α = 0.5 a second order paraboloid of revolution, **(C)** α = 1 a circular cone, and **(D)** α = 1.5 a hyperboloid of revolution.

The tapering mode α is a dimensionless parameter that describes the shape of a slender structure (Figure 2), which is, in our case, the petiole. Calculations of the tapering mode were based on the formulae published by Caliaro et al. (2013). First, we calculated the equivalent radius to account for non-perfectly circular cross-sections:


(3) r=radaxial3⋅rlateral4

where *r*_*adaxial*_ is the radius in the adaxial-abaxial direction (which is also the bending direction in which the force acts) and *r*_*lateral*_ is the radius in the lateral direction (perpendicular to the bending direction). The radii *r*_*adaxial*_ and *r*_*lateral*_ were derived from the corresponding measured diameters. Thereafter, the tapering mode α was derived based on the equation:


(4) $$\alpha =\frac{log\left(\frac{r\left(x\right)-r_{apical}}{r_{basal}-r_{apical}}\right)}{log\left(\frac{L-x}{L}\right)}$$

with *r(x)* being the equivalent radius at the distance *x* from the basal end of the petiole, *r*_*apical*_ being the equivalent radius of the apical end (*x* = *L*), *r*_*basal*_ being the equivalent radius of the basal end (*x* = 0) and *L* being the length of the petiole. The numerator was plotted against the denominator and the slope of the linear regression represented the tapering mode α.

### Anatomy and Histology

After mechanical testing, we divided the petioles into thirds, with 1 cm of the basal, middle and apical part of each petiole being frozen onto a metal sample holder by means of a freezing solution (Tissue-Tek O.C.T. Compound, Sakura Finetek Japan Co., Tokyo, Japan). Transverse thin sections with a thickness of 100 μm were cut on a rotatory cryotome (MEV, SLEE medical, Mainz, Germany). After bleaching the sections (20% eau de Javel), we immersed them in a 0.05% w/v solution of toluidine blue O, which stained non-lignified tissue red-purple and lignified tissue blue to dark violet (Sakai, 1973). The stained sections were imaged via an Olympus BX61 microscope (Olympus, Tokyo, Japan) equipped with a CP71 camera module. We determined the axial second moment of area *I* and polar second moment of area *J* for each section by using the BoneJ2 Plugin (Version 6.1.0) (Doube et al., 2010) provided in Fiji software (ImageJ Version 1.52p) (Schindelin et al., 2012). These geometric variables describe the influence of the cross-sectional shape of a specimen on its mechanical behavior, for example under bending (*I*) or torsional loads (*J*). The higher these geometrical characteristics are, the more the geometries resist mechanical loads/deformations.

**FIGURE 3:**
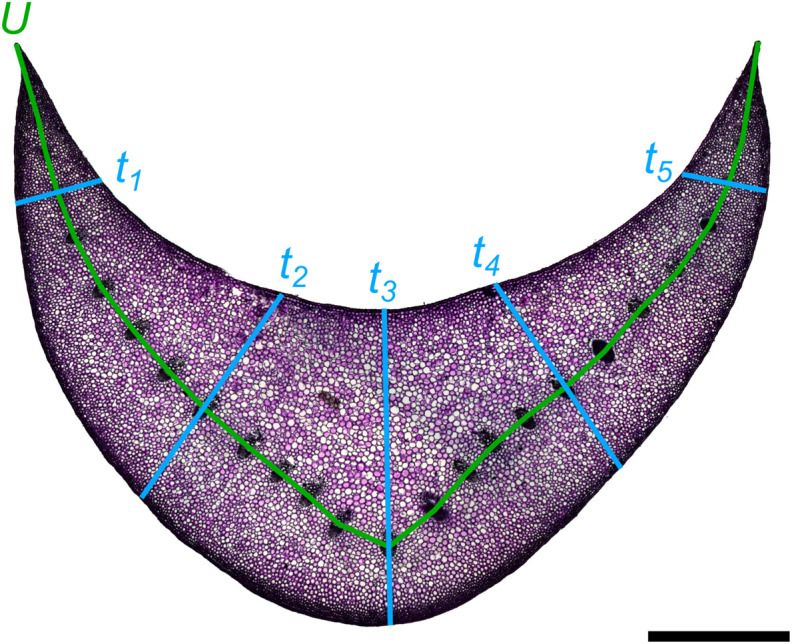
Exemplary cross-section of the petiole of *Hosta* x *tardiana* ‘El Niño’ with five measuring points for the thicknesses (*t*_1_–*t*_5_) and the median line *U*. The scale bar is 2 mm.

Another variable useful for analyzing the influence of the cross-sectional geometry on the torsional behavior is the torsion constant *K*. *K* is part of the following equation expressing torsional rigidity:


(5) $$\theta =\frac{T\cdot L}{K\cdot G}$$

where θ is the angle of twist in radians, *T* the twisting moment, *L* the length of the bar and *G* the torsional modulus. *K* is equal to the polar second moment of area *J*, provided that the cross-section is perfectly circular. For other cross-sectional geometries, however, *K* is smaller than *J* and, in some cases, is only a small fraction of it (Young et al., 2002). Therefore, different equations are needed for the various cross-sectional geometries. All equations have been taken from **Table 10.1** of Young et al. (2002). The petioles of *C. bicolor* and *P. peperomioides* are almost circular and have therefore been assumed to be circles; the following equation can thus be used to calculate *K*, which, in this case, equals *J*:


(6) $$K_{circle}=\frac{1}{2}\cdot \pi \cdot r^{4}$$

with *r* being the radius of the circle. The petioles of *H. alternata* are elliptical and, therefore, *K* has been calculated according to the following equation:


(7) Kellipse=π⋅radaxial3⋅rlateral3radaxial2+rlateral2

where *r*_*adaxial*_ is the radius in adaxial-abaxial direction and *r*_*lateral*_ the radius in lateral direction of the ellipse. For the U-profile of the petioles of *H. tardiana*, *K* has been calculated as follows:


(8) $$K_{U-profile}=\frac{1}{3}\cdot U\cdot t^{3}$$

with *U* being the length of the median line through the U-profile and *t* the thickness of the U-profile. However, this formula is limited to a uniform thickness of the U-profile, which is not the case with the cross-sectional geometry of the petioles of *Hosta* x *tardiana* ‘El Niño’. Therefore, we measured the thickness for each cross-section, always at the same five points of the cross-section, and calculated the mean thickness (Figure 3). This mean value was then used as *t* in the formula. The median line *U* was placed through the vascular bundles in each cross-section (Figure 3).

### Biomechanics

Petiole samples were glued basally into 3D-printed PLA (polylactide) clamps by using ethyl cyanoacrylate as the adhesive (LOCTITE^®^ 401, Henkel AG & Co., KGaA, Düsseldorf, Germany). The clamps were fixed within a custom-built uniaxial measurement device and two-point bending tests were performed. The petioles were always bent in the abaxial direction. The device was equipped with a 1 N force sensor (Burster 8510– 5001, Burster Präzisionstechnik GmbH & Co., KG, Gernsbach, Germany) with a data acquisition rate of one value per 0.09 mm deflection. Then the apical ends were glued into the PLA clamps and the samples were fixed into the custom-build manual torsional test device (Gallenmüller et al., 2001). Torque was manually applied by a spring-loaded cylinder (spring constant *c* = 0.006 Nm^–1^) and the resulting angles were recorded. In order to evaluate the structural resistance of the specimens to the mechanical stresses/deformations (bending and torsion), the flexural rigidity *EI* and torsional rigidity *GK* were calculated according to:


(9) EI=Lreal33⋅bbending

(10) $$GK=\frac{L_{real}}{b_{torsion}}$$

where *L*_*real*_ is the sample length, *b*_*bending*_ the slope in the displacement-force diagram and *b_*to*__*rsion*_* the slope in the angular deflection-torque diagram. We calculated the torsional rigidity *GK* by using the torsion constant *K*, which is valid for cross-sections of any geometry, unlike the polar second moment of area *J*, which is valid only for circular cross-sections. Based on Equations (9) and (10) and the approach of Caliaro et al. (2013), we calculated the elastic modulus *E*and torsional modulus *G* taking into consideration the tapering mode:


(11) E=Lreal33⋅Ibasal⋅bbending⋅(rbasalrapical)α

(12) $$G=\frac{L_{real}}{K_{basal}\cdot b_{torsion}}\cdot {\left(\frac{r_{basal}}{r_{apical}}\right)}^{\alpha }$$

with *I*_*basal*_ being the axial second moment of area at the basal end of the petiole, *r*_*basal*_ the equivalent radius of the petiole at the basal end, *r*_*apical*_ the equivalent radius of the petiole at the apical end, α the tapering mode and *K*_*basal*_ the torsion constant at the basal end of the petiole. *E* and *G* describe the resistance of the material on bending or torsional deformations.

The safety factor *SF* describes the multiple by which the structures can carry more than their actual static load. For the petioles, the *SF* was calculated as the ratio between the critical length (*L*_*max*_) and the real length (*L*_*real*_):


(13) $$SF=\frac{L_{max}}{L_{real}}$$

The challenge of the calculation of the critical length *L*_*max*_ at which a slender and vertically oriented structure (pole) buckles was first solved analytically for selected pole forms and a continuous mass distribution along the poles without additional top load (Greenhill, 1881). Speck (1994) developed a formula based on the approach of Greenhill (1881) who took into consideration a top load in addition to a mass continuously distributed along the poles. The formula of Speck (1994) was used to calculate *L*_*max*_ for the peltate leaves of *C. bicolor* and *P. peperomioides*, whose nearly vertically oriented petioles bear the lamina as a top load:


(14) Lmax=(π4)23⋅(2α+1)13⋅(E⋅rbasal2(π216⋅(2α+1)c3+(2α+1)⋅FlaminaFpetiole)⋅γ)13

where *c* is a shape factor depending on the tapering mode and weight distribution along the petiole (*c* = 1.96 for *C. bicolor* and *P. peperomioides*), *F*_*lamina*_ is the top load caused by the weight of the lamina, *F*_*petiole*_ is the load induced by the self-weight of the petiole and γ is the specific weight of the petiole.

However, none of these approaches, which are all based on Euler’s critical buckling length calculations for slender upright poles, is suitable for calculating the critical buckling length *L*_*max*_ of horizontally oriented plant structures, such as the petioles of *H. tardiana* and *H. alternata*. For this calculation, we considered horizontally oriented petioles as cantilever beams clamped on one side. We then considered the deflection δ of these beams under two different loading scenarios. To account for the deflection attributable to the self-weight of the petiole δ_*petiole*_, we used the following conventional formula for a (continuously) distributed load over the petiole:


(15) δpetiole=Fpetiole⋅Lreal38⋅EI

In addition, we calculated the deflection caused by the weight of the lamina as a top load:


(16) δlamina=Flamina⋅Lreal33⋅EI

The total deflection δ was calculated by summing up the individual deflections:


(17) δ=Fpetiole⋅Lreal38⋅EI+Flamina⋅Lreal33⋅EI

The solution of Equation (17) according to the length of the petiole yielded:


(18) $$L_{real}=\sqrt[{3}]{\frac{24\cdot EI\cdot \delta }{3\cdot F_{petiole}+8\cdot F_{lamina}}}$$

Finally, we made the assumption that a deflection δ equal to the length of the petiole *L*_*real*_ will inevitably lead to a buckling failure. Thus, if *L*_*real*_ is substituted for δ in Equation (18), the critical length *L*_*max*_ is obtained as follows:


(19) $$L_{max}=\sqrt[{3}]{\frac{24\cdot EI\cdot L_{real}}{3\cdot F_{petiole}+8\cdot F_{lamina}}}$$

However, in formula (19), the tapering mode α is not taken into account. Since we found tapered petioles in all the species studied, the tapering mode α was included in the flexural rigidity *EI*, similar to the approach given by Caliaro et al. (2013) for the elastic modulus *E* in Equation (11). The first term is the conventional equation for calculating the elastic modulus in two-point bending tests, with the assumption of a constant axial second moment of area *I*. The tapering mode α is included via the added second term, which incorporates a change in the axial second moment of area *I* based on its value at the base of the petiole (*I*_*basal*_). Rearrangement of this equation according to *EI* yields an *EI*_*tapered*_ depending on the tapering mode:


(20) $$EI_{tapered}=EI\cdot {\left(\frac{r_{apical}}{r_{basal}}\right)}^{\alpha }$$

Finally, we substituted *EI*_*tapered*_ for *EI* in Equation (19) and obtained the critical length *L*_*max*_ of a horizontally oriented petiole taking into account the tapering mode:


(21) $$L_{max}=\sqrt[{3}]{\frac{24\cdot EI\cdot {\left(\frac{r_{apical}}{r_{basal}}\right)}^{\alpha }\cdot L_{real}}{3\cdot F_{petiole}+8\cdot F_{lamina}}}$$

This formula was used to calculate *L*_*max*_ for *H. tardiana* and *H. alternata*.

### Statistical Analysis

All raw data are included in Supplementary Table 1. We used the software *GNU R* 4.0.0 for statistical analyses (R Core Team, 2021). The data were tested for normal distribution (Shapiro-Wilk test) and for homoscedasticity of variances (Levene test). Since all data are non-normally distributed, we present median values with corresponding interquartile ranges in brackets (IQR). We tested for significance at a significance level of 5% and performed Kruskal-Wallis tests together with Mann-Whitney-U *post hoc* tests (with *p*-value adjustments according to Holm, 1979) for unpaired data (*p*-values are given in Supplementary Table 2).

## Results

To analyze the form-function relationship of the selected petioles, we focused on four key aspects: geometry, size, shape and biomechanics. In Table 1, we present our results as median values and corresponding IQR (in brackets). In addition, the results of the statistical analyses (*p*-values) are provided in Supplementary Table 2.

**TABLE 1:** Descriptive statistics of the variables of the petioles of *Hosta* ×* tardiana* ‘El Niño’, *Caladium bicolor*, *Hemigraphis alternata*, and *Pilea peperomioides* giving an overall view of all numerical findings.

	Body plan	Monocotyledons		Dicotyledons		

	Configuration	2D	3D	2D	3D	

	Species	*Hosta* x *tardiana* ‘El Niño’	*Caladium bicolor*	*Hemigraphis alternata*	*Pilea peperomioides*	

Variable	Description	Median (IQR)	Median (IQR)	Median (IQR)	Median (IQR)	*n*
**Geometry**						
	Cross-sectional geometry	*U-profile*	*Circle*	*Ellipse*	*Circle*	
**Size**						
*I*_*basal*_[mm^4^]	Axial second moment of area at the petiole base	145.84 (53.86)	198.02 (77.63)	1.64 (0.54)	7.96 (5.22)	25
*J*_*basal*_ [mm^4^]	Polar second moment of area at the petiole base	513.74 (175.56)	358.12 (158.04)	4.31 (1.16)	16.37 (12.64)	25
*K*_*basal*_ [mm^4^]	Torsion constant at the petiole base	136.43 (36.31)	341.94 (95.80)	2.65 (0.89)	11.92 (8.75)	25
**Shape**						
*AR* [-]	Aspect ratio (Langer et al., 2021)	1.13 (0.13)	0.95 (0.09)	1.22 (0.09)	1.05 (0.08)	25
*I/J* [-]	Ratio of axial to polar second moment of area (Langer et al., 2021)	0.33 (0.05)	0.53 (0.05)	0.40 (0.03)	0.47 (0.04)	25
*I/K* [-]	Ratio of axial second moment of area and torsion constant	1.08 (0.24)	0.58 (0.24)	0.60 (0.13)	0.69 (0.10)	25
α [-]	Tapering mode (Langer et al., 2021)	1.47 (0.40)	0.91 (0.15)	1.36 (0.57)	1.18 (0.54)	25
*WR* [-]	Weight ratio of lamina to petiole	0.99 (0.18)	0.59 (0.17)	7.50 (6.07)	2.75 (0.68)	25
**Biomechanics**						
*EI/GK* [-]	Twist-to-bend ratio	23.66 (6.85)	39.19 (15.01)	11.47 (3.50)	13.30 (4.09)	25
*EI* [Nmm^2^]	Flexural rigidity	8,938.77(5,252.51)	17,463.92(6,456.76)	210.98 (93.79)	503.88 (346.27)	25
*GK* [Nmm^2^]	Torsional rigidity	377.76 (98.54)	469.35 (159.96)	17.79 (3.88)	41.25 (27.16)	25
*E/G* [-]	Modulus ratio	22.14 (7.69)	63.73 (44.15)	18.20 (7.10)	19.73 (5.99)	25
*E* [MPa]	Bending elastic modulus	132.88 (43.04)	192.19 (125.39)	171.26 (65.24)	110.25 (29.16)	25
*G* [MPa]	Torsional modulus	6.37 (2.03)	2.90 (0.59)	8.46 (2.49)	5.74 (2.90)	25
*SF* [-]	Safety factor	2.35 (0.50)	1.66 (0.25)	2.94 (0.59)	2.13 (0.43)	25

*Results are presented as median with interquartile range (=IQR) in brackets; n corresponds to the sample size.*

### Geometry and Anatomy

**FIGURE 4:**
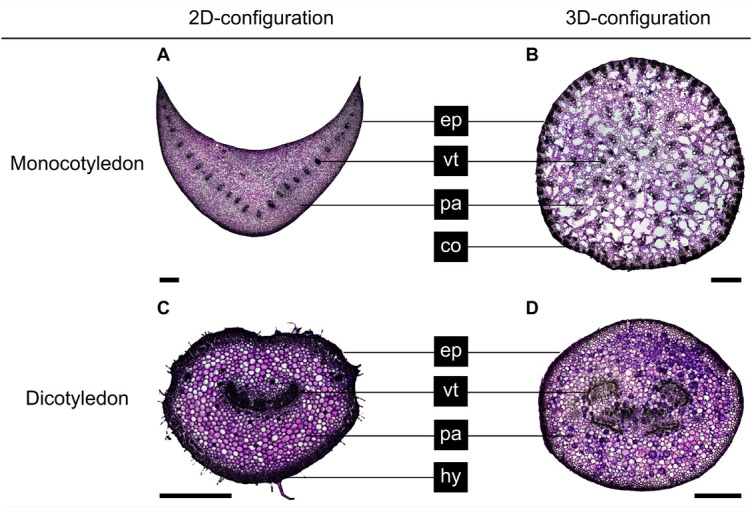
Basal cross-sections of **(A)** *Hosta* ×* tardiana* ‘El Niño’, **(B)** *Caladium bicolor*, **(C)** *Hemigraphis alternata*, and **(D)** *Pilea peperomioides* stained with toluidine blue O. Epidermis (ep), vascular tissue (vt), parenchyma (pa), collenchyma (co), and hypodermis (hy) are shown. Scale bars are 1 mm.

The cross-sectional geometries of the petiole types varied from circular (*C. bicolor*, *P. peperomioides)* to elliptical (*H. alternata*) or were U-profiled (*H. tardiana*) (Figure 4). In addition to the epidermis, the parenchyma and the vascular tissue, we found strengthening tissues: *C. bicolor* (Figure 4B) has individual strands of collenchyma fibers in the periphery of the cross-section, whereas, in *H. alternata* (Figure 4C), the strengthening tissue forms a peripheral hypodermal ring.

### Size

The size variables represented by the axial (*I*) and polar (*J*) second moment of area and torsion constant (*K*) of the monocotyledonous petioles were significantly higher than those of the dicotyledonous petioles. Thus, in terms of the size-dependent variables *I*, *J*, and *K*, the petioles of the monocotyledons were more resistant to deformations by bending and torsional loads. Within the dicotyledons, all size variables of *P. peperomioides* were significantly higher than those of *H. alternata*. Within the monocotyledons, *C. bicolor* exhibited significantly higher values for *I* and *K*, whereas *H. tardiana* had a significantly higher *J*.

### Shape

**FIGURE 5:**
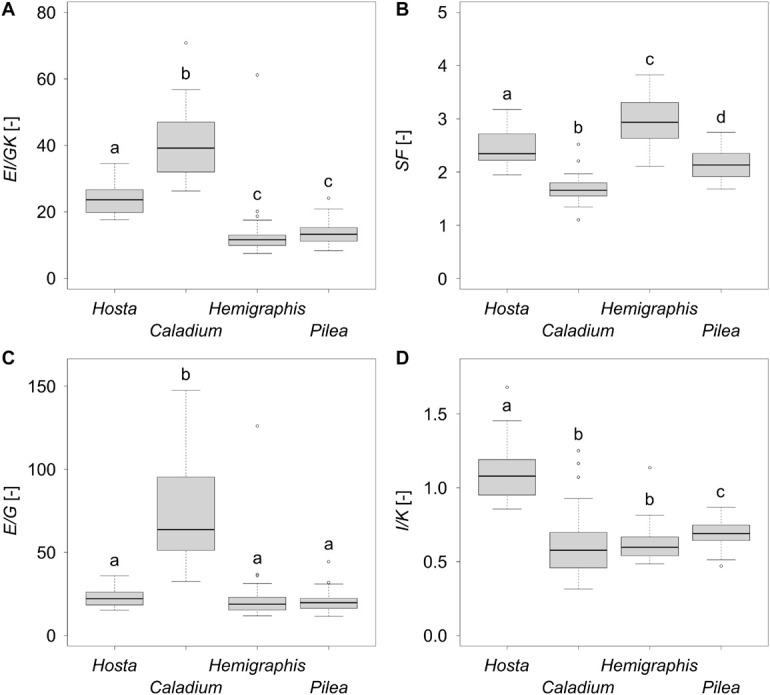
Boxplots of **(A)** the twist-to-bend ratio *EI/GK*, **(B)** the safety factor *SF*, **(C)** the ratio of the elastic to the torsional modulus *E/G*, and **(D)** the ratio of the axial second moment of area to the torsion constant *I/K* of 25 leaves each of the species *Hosta* x *tardiana* ‘El Niño’, *Caladium bicolor*, *Hemigraphis alternata*, and *Pilea peperomioides*. Significant differences (*p*-value < 0.05) are indicated by lower case letters.

The circular geometry of the petioles of *C. bicolor* and *P. peperomioides* was confirmed numerically by their respective aspect ratios of *AR* ≈ 1.0 and their ratios of the second moments of area of *I/J* ≈ 0.5. In contrast, we found *AR* > 1.0 and *I/J* < 0.5 for the elliptical geometry of *H. alternata* and the U-profile of *H. tardiana*. The *I/K* values of the circular and elliptical geometries ranged between 0.58 and 0.69 and differed significantly from the U-profile of *H. tardiana* with 1.08 (Figure 5D). Thus, *H. tardiana* has the comparably smallest *K* in relation to its *I*. The weight ratio *WR* of the lamina and petiole was balanced in *H. tardiana*, whereas the lamina of *C. bicolor* only weighed approx. 2/3 the weight of the petiole. In comparison, the lamina of dicotyledons *H. alternata* and *P. peperomioides* weighed several times the weight of the petiole, showing that the petioles of the dicotyledons carried, in relation to their own weight, significantly more weight than those of the monocotyledons. The petioles of *C. bicolor* were linearly tapered (α ≈ 1) and differed significantly from the other investigated species. The petioles of the other species were more hyperbolically tapered (α > 1), i.e., their cross-sections tapered faster in the apical direction than those of *C. bicolor*.

### Biomechanics

The bending elastic moduli *E* of all species were between 100 and 200 MPa. In particular, *E* of *P. peperomioides* was significantly smaller than those of the other species. Furthermore, *E* of *H. tardiana* was significantly smaller than that of *C. bicolor*. Therefore, with respect to the material property *E*, *C. bicolor* and *H. alternata* were more resistant against deformation by bending loads. Although the torsional moduli *G* were within a single-digit megapascal range, all torsional moduli *G* differed significantly with the exception of *H. tardiana* and *P. peperomioides*. *Caladium bicolor* had the lowest *G*, i.e., it exhibited the lowest material-based resistance to torsion. In contrast, *H. alternata* had the highest *G* and thus the highest material-based resistance to torsion. The median *E/G* for all species was about 20, except for *C. bicolor*, which had a significantly higher *E/G* of 63 (Figure 5C). The flexural and torsional rigidities *EI* and *GK* of the monocotyledonous petioles were significantly higher than those of the dicotyledonous petioles, which suggested that the monocotyledonous petioles were structurally better equipped to withstand bending and torsional loads. All twist-to-bend ratios *EI/GK* were above 10, showing that the petioles of all species studied were much easier to twist than to bend. However, the *EI/GK* ratio of the monocotyledons were significantly higher than those of the dicotyledons (Figure 5A). Among the studied monocotyledons, *C. bicolor* had the highest *EI/GK* ratio. The twist-to-bend ratios of the dicotyledons did not differ significantly. All safety factors showed significant differences with their medians ranging between 1.66 and 2.94 (Figure 5B).

## Discussion

As almost all variables studied showed significant differences, the aim of our screening process to select four different types of petioles as models for the two body plans and two configurations was successful. Nevertheless, we found similarities and dissimilarities between them. Generally, the high flexural rigidity of the petioles guarantees an optimal alignment of the lamina to sun light. This applies to loads arising from their own weight and to additional loads such as wind, rain, and snow. Streamlining in wind, however, is achieved both by the bending and twisting of the petioles and by the folding of the lamina (Vogel, 1989).

### High Twist-to-Bend Ratios

In general, high flexural rigidity (*EI*) combined with low torsional rigidity (*GK*) results in high twist-to-bend ratios (*EI/GK*). With a viewpoint on geometry, high twist-to-bend ratios thus result from high values of the axial second moment of area *I* compared with the torsion constant *K* (*I/K* > 1.0); in our study, this is true for the U-profile of the petiole of *H. tardiana*. With a focus on material properties, high twist-to-bend ratios can be the result of a high elastic modulus *E* and a relatively low torsional modulus *G* (*E/G* > > 1.0); this holds true for most plant organs (Niklas, 1999).

Since the polar second moment of area *J* is the sum of the perpendicular axial second moments of area *I* in x- and y-direction, the ratio of *I_*x*_/J* and *I_*y*_/J* can never exceed 1.0. For a perfect circular cross-section, *I/J* = 0.5. In this case, *K* is equal to *J* because circular cross-sections do not warp under torsional loading (Young et al., 2002; Etnier, 2003; Gere, 2004). However, all other geometries undergo warping under torsional loading and should be characterized by the torsion constant *K*, which is smaller than *J* and, thus, markedly influences *GK* and *EI/GK*. For example, *K* of the U-profile of the petiole of *H. tardiana* is 3.77 times smaller than the corresponding *J*. Furthermore, *K* of the ellipse of the petiole of *H. alternata* is 1.63 times smaller than *J*. Even for the almost circular cross-sections of *C. bicolor* and *P. peperomioides*, *K* is 1.05 and 1.37 smaller than *J* because biological samples are never perfectly circular. Since the literature values mostly refer to *J* and not to *K*, we will use *EI/GJ* for the comparative discussion on the twist-to-bend ratio. However, these twist-to-bend ratios from the literature might be underestimated, because *EI/GJ* < *EI/GK*.

**FIGURE 6:**
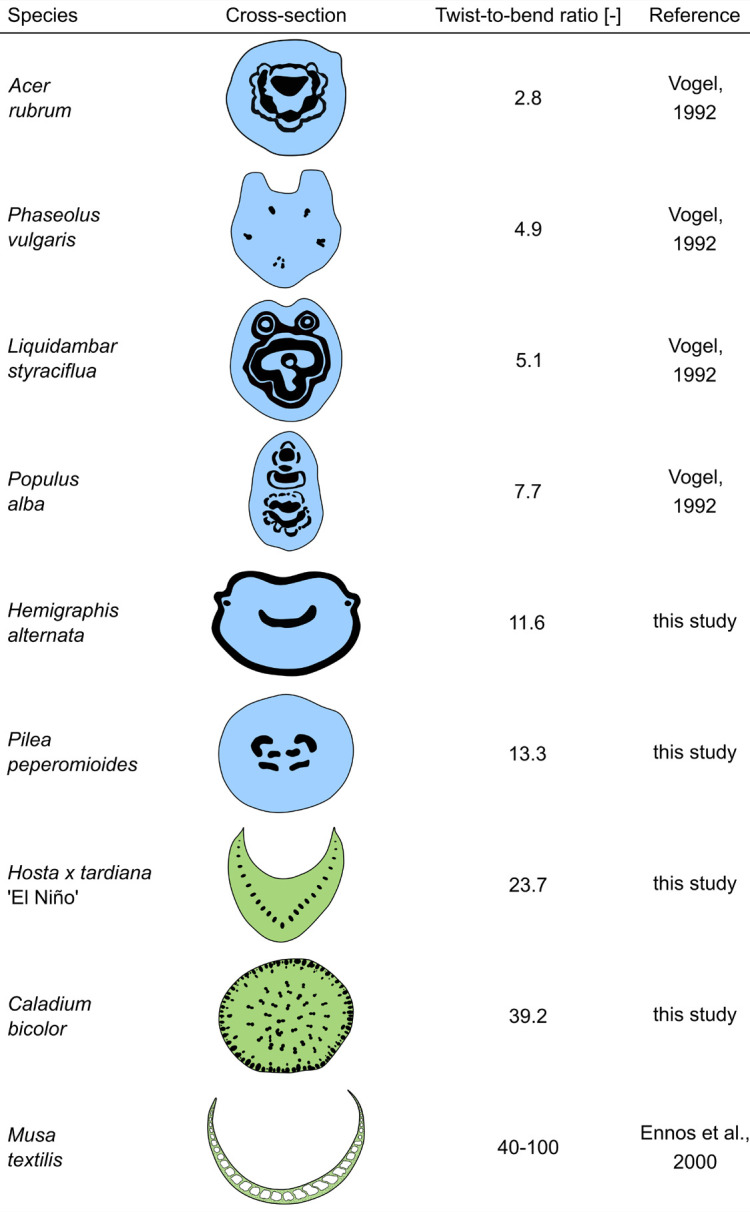
Schematic drawings of the cross-sectional geometries and strengthening tissues (black) of various petioles and their corresponding twist-to-bend ratios. The cross-sectional schematics are colored in blue for dicotyledons and in green for monocotyledons.

In the literature, exceptionally high twist-to-bend ratios (*EI/GJ*) are described for the U-profiled petioles of *Musa textilis* (Figure 6) and the triangular flower stalks of *Carex pendula*. Leaves of *Musa textilis* have long petioles with a pronounced taper and two large lamina halves. The petioles are U-profiled, similar to those of *H. tardiana*, and reveal an inner and outer shell with fiber-reinforced radial strands. This structure makes the petiole 40- to 100-fold stiffer in bending than in torsion (Ennos et al., 2000). In other words, the petiole is stiff enough to prevent the leaf from bending downwards and flexible enough to support streamlining by torsion. Peak values of up to 400 have been found in the triangular cross-sections of the flower stalk of *Carex pendula* (Speck et al., 2020). The high twist-to-bend ratios derive from high ratios of the bending elastic modulus and torsional modulus (*E/G*) with median values of 438. Flower stalks and leaf petioles, as all plant axes, consist of several tissues, and not only the presence of these tissues but also the tissue arrangement and distribution in the plant axes is important for the mechanical properties. This holds true especially for strengthening tissues like vascular bundles, collenchyma, and sclerenchyma. In the case of the *C. pendula* flower stalks the peripheral arrangement of the individual sclerenchyma strands, in combination with the high elastic modulus of sclerenchyma, is predominantly responsible for the high flexural rigidity of the flower stalks (Niklas, 1999; Wolff-Vorbeck et al., 2021). On the other hand, according to Wolff-Vorbeck et al. (2021), the reinforcement by not connected individual sclerenchyma strands only moderately increases the torsional rigidity of the entire stalk. While the bending stiffness remains almost constant with an increasing number of peripheral sclerenchyma strands, the torsional rigidity shows a minimum and the twist-to-bend ratio a maximum at 49 sclerenchyma strands. The results found for flower stalks of *C. pendula* can be transferred to the petioles of *C. bicolor* showing a similar arrangement of individual strands of strengthening tissues in the periphery. This applies even though the petioles of *C. bicolor* have a circular cross-section and possess collenchyma fibers instead of sclerenchyma fibers.

In this context, the different biomechanical properties of the strengthening tissues play a major role. Vascular bundles typically exhibit elastic moduli ranging from 30 to 840 MPa. Sclerenchyma, a dead and thick-walled strengthening tissue, is almost as stiff as wood under bending and twisting (Niklas, 1999). Sclerenchyma fibers, as found in *C. pendula*, have elastic moduli between 22,600 and 45,000 MPa (Niklas, 1992; Speck and Schmitt, 1992; Speck et al., 2018; Bold et al., 2020; Speck and Speck, 2021). In contrast, collenchyma, as present in *C. bicolor* petioles, is a living, hydrostatic and pronounced viscoelastic tissue, which is thus capable of large extensions, but at the same time restores itself after the removal of the external forces (Niklas, 1999). Therefore, the elastic modulus of collenchyma is markedly lower and ranges between 1,000 and 2,600 MPa (Ambronn, 1881; Niklas, 1992; Speck and Schmitt, 1992; Speck and Speck, 2021). Both sclerenchyma and collenchyma are important strengthening tissues in all types of plant axes. They resist bending and torsional loads through high stiffness and/or absorb these loads through their viscoelasticity.

### Tapering Mode

The petiole can be considered as a horizontal cantilever or vertical pole that is fixed at one end and in which the load of the lamina is applied to the free end. Thus, the larger the tapering mode, the more weight can be saved in the apical region, whereby its own weight and the acting leverage (in the horizontal orientation) are minimized (Figure 2). In this study, the applied weight of the lamina differs markedly and ranges from 0.6 to 7.5 times the petiole weight. Interestingly, the petiole of *H. alternata* has the smallest values for flexural rigidity but carries the highest top load in relation to its weight. In this context, Silk et al. (1982) have pointed out that tapered axes apically have a lower flexural rigidity, which leads to a bending at the tip and a reduction of the moment arm produced by the top load (e.g., lamina, flowers, fruits). The calculation of the tapering mode α is also essential for another reason. For calculations of mechanical properties, the tapering mode is of particular importance because its omission can lead to their considerably overestimation. For the petioles of *Caladium bicolor* “Candyland” Caliaro et al. (2013) have determined a tapering mode of 0.82. By considering this tapering mode, the flexural rigidity decreases to 59% of the value without the taper. Similarly, the flower stalks of *C. pendula* have a tapering mode of 1.37, which reduces the flexural rigidity to 88% of the value without the taper (Speck et al., 2020).

### Leaves With 2D-Configuration

Leaves with a 2D-configuration, such as those of *H. tardiana* and *H. alternata*, are mostly horizontally oriented. In this context, elliptical cross-sections with a groove, as in *H. alternata*, *Phaseolus vulga*ris and *Liquidambar styraciflua*, or U-profiles, as in *H. tardiana* and *Musa textilis*, are advantageous as they are resistant to downward bending. In addition, these cross-sections allow for high torsional flexibility, making them well suited for handling wind loads through streamlining (Figure 6; Vogel, 1992; Ennos et al., 2000; Wolff-Vorbeck et al., 2019). A contrary influence is the closed ring of peripheral strengthening tissue, i.e., the hypodermis of *H. alternata*, which increases flexural and torsional rigidities (Figure 4; Niklas, 1999).

### Leaves With 3D-Configuration

The petioles of leaves with a 3D-configuration grow vertically but are sometimes slightly inclined not only as a result of growth processes, but also because of the eccentric connection of the petiole to the lamina and the associated slightly asymmetric weight distribution. Peltate leaves often possess petioles with an almost circular cross-section, as found in *C. bicolor* and *P. peperomioides*, which causes no preferred or disadvantaged bending force direction attributable to the apical load caused by the lamina (Vogel, 1992; Sacher et al., 2019). The petioles of *C. bicolor* show a low *I/K* ratio but their *E/G* ratio is more than three times higher than that of the other petioles tested resulting in high twist-to-bend ratios (Figure 6). The high *E/G* ratio of *C. bicolor* petioles is due to the fact that they have in median the highest *E* and lowest *G* of the species studied. The high *E* can be explained by the already described peripherally arranged strengthening tissue in the form of collenchyma fibers. The low torsional rigidity can be attributed to the arrangement of the strengthening tissues (vascular and collenchyma bundles) in separate individual not connected strands. This tissue arrangement is quite flexible in torsion, as mentioned earlier, compared to closed rings of strengthening tissues as found in *H. alternata* (Niklas, 1999; Ennos et al., 2000; Wolff-Vorbeck et al., 2021).

Overall, the material properties *E* and *G* of all the species studied are of a similar magnitude and have typical values for organs of non-lignified herbaceous plants (Speck and Speck, 2021). With regard to the monocotyledons studied, the high values of *I*, *J*, and *K* result from their broader petioles and lead to significant differences in flexural and torsional rigidities. The high flexural rigidities of these monocotyledons, in turn, translate into their significantly higher twist-to-bend ratios (Figure 6).

### Safety Factors

The safety factors ranging between 1.66 and 2.94 indicate that each petiole can carry about twice to triple the static load of the leaf, leaving enough margin for additional loads such as wind or rain. This agrees well with the reported safety factor of biological structures, such as the flower stalk of the monocotyledonous *Allium sativum* with a safety factor of 1.85 ± 0.29 (Niklas, 1990) or the stems of the dicotyledonous *Populus tremuloides* with an average safety factor of 2.3 (King and Loucks, 1978). In contrast, large old record-sized trees, when only their own weight is considered, are mechanically “overbuilt” and have a safety factor of over 4 (McMahon, 1973; King and Loucks, 1978; Niklas, 1992). In herbaceous plants, however, the safety factor depends on the turgescence of the tissues. Fully turgescent peduncles of the dicotyledonous *Gerbera jamesonii* ‘Nuance’ have a safety factor of 1.42. This is different in wilted peduncles, which have a safety factor of 0.95 and cause pronounced drooping of their flower heads (Lehmann et al., 2019).

## Conclusion

With respect to our scientific question of “How do cross-sectional geometry, sizes and shapes of petioles influence their twist-to-bend ratio and safety factor?” we can make some general statements:

• High twist-to-bend ratios allow the petiole to be stiff enough to withstand bending loads caused by the self-weight of the leaf and to be flexible enough to twist away from damaging influences such as wind loads.
• Strengthening tissue in the periphery (e.g., fibers, hypodermis) increases flexural rigidity.
• Closed peripheral rings of strengthening tissue (e.g., hypodermis) markedly increase torsional rigidity. Individual strands of strengthening tissue (e.g., fiber strands) do not markedly increase torsional rigidity. Adaxial grooves (e.g., U-profile) decrease torsional rigidity.
• With the exception of perfect circular cross-sections, the polar second moment of area *J* is considerably larger than the torsion constant *K*, the latter value having been calculated by taking into account warping under torsional loading.
• Since *K* < *J*, it follows that *EI/GJ* < *EI/GK*, which leads to an underestimation of the “real twist-to-bend ratios” of structures with cross-sections exhibiting warping, if *J* is used for calculating the twist-to-bend ratio.
• High twist-to-bend ratios can be geometry-related if *I/K* > 1.0, which is rare, and/or material-related if *E/G* > > 1.0, which holds true for most plant organs.
• The safety factor, here defined as the ratio of maximal length to real length, describes the extent to which the petiole can support more than its own weight.
• Equations to calculate the safety factor are now available for horizontally and vertically oriented leaves.
• The safety factor of herbaceous plants depends on their turgor.

In addition to the above-mentioned general statement, our comparative morphological, anatomical and biomechanical investigations of four petiole types have revealed further dissimilarities and similarities:

• The petioles differ in most of the variables that we have measured and calculated, namely geometry, shape, size, and mechanics.
• The twist-to-bend ratios of the petioles of the monocotyledons *H. tardiana* and *C. bicolor* are significantly higher than those of the dicotyledons *H. alternata* and *P. peperomioides*.
• Dependent on the cross-sectional geometry of the petioles, the torsion constant *K* is smaller than *J* as follows: 3.77 times smaller for the U-profile of *H. tardiana*, 1.63 times smaller for the ellipse of *H. alternata*, 1.37 times smaller for the circle of *P. peperomioides* and 1.05 times smaller for the circle of *C. bicolor*.
• The U-profiles of *H. tardiana* is the only one with an *I/K* > 1.0.
• The *E/G* of *C. bicolor* is three times as high as that of the other petioles.
• The safety factors strongly indicate that each of the petioles studied can support about double to triple the leaf’s own weight, with sufficient tolerance for additional loads such as wind.

In conclusion, our results show that high twist-to-bend ratios, i.e., high flexural rigidity and low torsional rigidity, can be achieved by geometrical-based (U-profile) or by material-based (high *E/G* ratio) conditions. On the other hand, some plant axes show low twist-to-bend ratios, i.e., low flexural rigidity and high torsional rigidity, which can even be smaller than 1 in special cases. For example, cross-sections with a peripheral closed ring of strengthening tissue show high torsional rigidity. These results for plant petioles and flower stalks can be transferred to other rod-shaped axes of plants and animals with different cross-sectional geometry. Furthermore, our findings may serve as inspiration for technical applications using rod-shaped axes. In the framework of a biomimetic approach, axes with high or low twist-to-bend ratios can be created by transferring our findings regarding the importance of cross-sectional geometry and arrangement of strengthening elements or ratios of material properties. Based on the results of the present study, the question arises what influence the cross-sectional geometry and/or arrangement of strengthening tissues have on the flexural and torsional rigidity and thus on the twist-to-bend ratio in general. A further systematic study on this topic using a modeling approach is in progress and will provide further information about the mechanical performance of plant axes and can also be a starting point for engineers to develop bioinspired solutions.

Moreover, our calculations revealed safety factors for herbaceous fully turgescent plants between 2 and 3. Interestingly, a safety factor between 1.2 and 3.0 is used in engineering, depending on the materials used and the particular application. In the case of a wilting plant, the safety factor decreases and can even fall below 1, meaning that the structure will eventually fail. However, the turgor-dependent change of safety factors in herbaceous plants may be an inspiration for technical solutions, where a variation of mechanical properties with a concomitant change/adaptation of the safety factor depending on the phase of use might be beneficial.

## Data Availability Statement

The original contributions presented in the study are included in the article/Supplementary Material, further inquiries can be directed to the corresponding author/s.

## Author Contributions

ML, CM, TS, and OS designed the study, performed the biomechanical analyses, and wrote the final draft of the manuscript. MK and ML collected the data and performed the statistical analyses. ML and OS wrote the first draft of the manuscript. All authors contributed significantly to the intellectual content of the final draft, revised the article, approved the final version of the manuscript, and agree to be held responsible for the content therein.

## Conflict of Interest

The authors declare that the research was conducted in the absence of any commercial or financial relationships that could be construed as a potential conflict of interest.

## Publisher’s Note

All claims expressed in this article are solely those of the authors and do not necessarily represent those of their affiliated organizations, or those of the publisher, the editors and the reviewers. Any product that may be evaluated in this article, or claim that may be made by its manufacturer, is not guaranteed or endorsed by the publisher.
