# Supplementary material for: Twist-to-Bend Ratios and Safety Factors of Petioles Having Various Geometries, Sizes and Shapes
Source: Front Plant Sci. 2021 Nov 11;12:765605. doi: 10.3389/fpls.2021.765605 (PMC8632552; doi:10.3389/fpls.2021.765605)
Supplement: Supplementary file 1 [file Data_Sheet_1.PDF]

Sample	*Hosta x tardiana* 'El Niño'	*Caladium bicolor*	*Hemigraphis alternata*	*Pilea peperomioides*
	[-]	[-]	[-]	[-]
1	1.12	0.58	0.63	0.78
2	1.22	0.50	0.49	0.63
3	0.87	0.41	0.73	0.52
4	0.98	0.48	0.49	0.64
5	1.09	0.48	1.14	0.51
6	0.96	0.70	0.81	0.53
7	0.95	0.58	0.49	0.67
8	0.94	0.38	0.51	0.79
9	0.86	0.46	0.72	0.87
10	1.08	1.25	0.56	0.66
11	1.68	0.70	0.53	0.75
12	0.92	0.68	0.63	0.70
13	0.87	0.50	0.63	0.75
14	0.96	0.31	0.60	0.69
15	1.28	1.16	0.67	0.64
16	1.14	0.38	0.56	0.47
17	1.19	0.93	0.71	0.86
18	1.07	0.50	0.67	0.65
19	1.06	0.60	0.63	0.65
20	1.08	0.58	0.55	0.72
21	1.45	0.41	0.56	0.69
22	0.95	0.72	0.53	0.81
23	1.15	1.07	0.65	0.73
24	1.19	0.69	0.59	0.65
25	1.25	0.38	0.54	0.81
**Median**	1.08	0.58	0.60	0.69
**IQR**	0.24	0.24	0.13	0.10

Weight ratio of lamina to petiole
WR

Sample	*Hosta x tardiana* 'El Niño'	*Caladium bicolor*	*Hemigraphis alternata*	*Pilea peperomioides*
	[-]	[-]	[-]	[-]
1	1.24	0.48	11.75	2.75
2	1.14	0.47	8.36	2.67
3	1.37	0.49	18.57	2.13
4	0.96	0.47	7.10	4.41
5	0.72	0.51	10.29	3.52
6	0.97	0.77	10.71	2.08
7	0.97	0.61	8.23	2.95
8	1.07	0.41	3.75	2.69
9	1.06	0.35	4.42	2.35
10	1.02	0.66	4.25	2.59
11	1.00	0.75	43.69	2.27
12	0.65	0.70	21.76	3.10
13	0.89	0.48	23.14	2.42
14	1.06	0.54	9.56	2.67
15	0.82	0.63	7.50	2.85
16	1.07	0.61	4.04	2.95
17	1.18	0.66	5.03	2.07
18	0.91	0.52	4.61	3.08
19	0.83	0.70	5.28	3.60
20	0.87	0.67	3.16	3.49
21	0.84	0.58	10.01	2.53
22	0.98	0.59	13.76	3.11
23	0.99	0.70	4.65	2.09
24	1.03	0.48	6.53	3.16
25	1.05	0.61	5.70	2.81
**Median**	0.99	0.59	7.50	2.75
**IQR**	0.18	0.17	6.07	0.68

Axial second moment of area of the basal petioles *I* $_{basal}$

**Sample**	***Hosta x tardiana* 'El Niño'**	***Caladium bicolor***	***Hemigraphis alternata***	***Pilea peperomioides***
	[mm^4]	[mm^4]	[mm^4]	[mm^4]
1	156.54	199.34	1.32	10.73
2	189.41	181.40	1.83	12.17
3	80.13	185.83	1.39	6.47
4	183.40	171.13	2.04	9.51
5	129.54	244.72	1.32	5.59
6	119.41	260.47	1.99	10.46
7	174.94	250.28	1.12	4.45
8	102.69	137.92	2.81	10.46
9	186.63	143.49	1.92	8.26
10	132.23	221.12	1.54	5.43
11	182.52	198.02	2.53	7.18
12	134.65	217.66	1.90	15.37
13	188.39	123.95	1.91	6.80
14	137.60	111.63	1.44	14.46
15	122.31	249.55	1.64	5.43
16	139.09	119.63	1.54	7.96
17	154.90	203.23	1.50	10.65
18	141.41	218.96	1.77	4.43
19	145.84	214.26	1.18	13.94
20	183.81	153.69	1.20	16.38
21	220.56	91.36	1.75	8.23
22	110.44	160.53	1.75	5.33
23	202.71	305.03	2.06	5.79
24	162.79	289.00	1.38	5.04
25	122.52	129.97	1.22	5.39
**Median**	145.84	198.02	1.64	7.96
**IQR**	53.86	77.63	0.54	5.22

Polar second moment of area of the basal petioles J_{basal}

**Sample**	***Hosta x tardiana* 'El Niño'**	***Caladium bicolor***	***Hemigraphis alternata***	***Pilea peperomioides***
	$[mm^4]$	$[mm^4]$	$[mm^4]$	$[mm^4]$
1	580.99	358.12	3.94	22.11
2	710.42	361.24	4.77	27.18
3	223.63	352.39	3.56	15.39
4	650.26	330.52	6.12	18.35
5	418.25	449.53	4.16	12.85
6	469.79	502.18	4.76	21.36
7	675.09	416.53	3.34	9.92
8	458.02	278.72	7.64	25.49
9	649.50	269.19	4.61	16.37
10	440.37	400.67	3.81	13.63
11	513.74	275.19	6.48	15.27
12	384.82	449.78	4.50	32.36
13	595.22	211.61	4.72	13.71
14	527.71	209.88	3.57	33.10
15	439.14	444.72	4.31	10.57
16	372.31	236.73	4.40	16.90
17	480.15	403.87	4.24	27.02
18	419.95	427.23	4.18	9.53
19	548.62	388.94	3.44	34.06
20	614.70	266.79	3.09	38.67
21	537.30	175.25	4.56	19.09
22	344.41	284.82	4.70	12.45
23	695.10	554.51	5.42	13.39
24	646.24	450.20	3.55	11.42
25	450.14	210.81	3.30	10.56
**Median**	513.74	358.12	4.31	16.37
**IQR**	175.56	158.04	1.16	12.64

Torsion constants K_{basal}

**Sample**	***Hosta x tardiana* 'El Niño'**	***Caladium bicolor***	***Hemigraphis alternata***	***Pilea peperomioides***
	[mm^4]	[mm^4]	[mm^4]	[mm^4]
1	140.15	343.47	2.10	13.75
2	155.66	362.06	3.75	19.25
3	92.51	451.99	1.91	12.52
4	187.50	353.96	4.19	14.94
5	119.35	508.66	1.16	10.90
6	124.73	370.89	2.45	19.81
7	184.95	433.92	2.31	6.61
8	109.05	358.28	5.52	13.24
9	218.01	313.41	2.68	9.52
10	122.03	176.86	2.76	8.17
11	108.69	283.89	4.79	9.60
12	146.97	319.43	3.01	22.08
13	216.90	246.85	3.02	9.10
14	143.54	354.46	2.41	20.93
15	95.23	214.43	2.46	8.44
16	122.38	313.94	2.75	16.92
17	130.13	219.31	2.11	12.35
18	132.33	438.51	2.65	6.81
19	137.66	359.55	1.87	21.34
20	170.43	266.26	2.19	22.64
21	151.78	220.18	3.15	11.92
22	116.10	223.45	3.31	6.58
23	176.60	284.80	3.17	7.94
24	136.43	421.52	2.34	7.73
25	98.01	341.94	2.26	6.65
**Median**	136.43	341.94	2.65	11.92
**IQR**	36.31	95.80	0.89	8.75

Twist-to-bend ratios *EI/GK*

**Sample**	***Hosta x tardiana* 'El Niño'**	***Caladium bicolor***	***Hemigraphis alternata***	***Pilea peperomioides***
	[-]	[-]	[-]	[-]
1	26.51	39.31	12.29	24.15
2	31.72	31.51	11.28	12.48
3	17.64	39.19	9.42	14.00
4	26.69	38.19	17.55	13.65
5	18.34	30.20	13.84	8.36
6	29.08	33.12	10.86	10.93
7	25.20	31.86	8.52	15.30
8	21.86	56.76	12.92	17.50
9	24.49	51.62	12.27	14.19
10	20.25	40.72	11.47	9.40
11	30.86	29.02	8.69	8.64
12	20.93	70.84	10.31	10.42
13	18.28	32.00	8.63	15.28
14	34.52	38.55	18.75	13.30
15	19.56	40.05	12.70	10.72
16	31.33	30.78	12.57	11.47
17	23.10	48.34	11.64	3.50
18	23.66	41.83	13.06	11.21
19	18.12	47.66	7.51	20.83
20	22.08	36.23	20.18	13.05
21	25.19	41.75	10.13	11.26
22	24.75	36.77	9.95	18.08
23	19.38	49.23	15.31	19.11
24	31.06	26.31	8.03	11.47
25	19.83	47.01	8.31	3.50
**Median**	23.66	39.19	11.47	13.30
**IQR**	6.85	15.01	3.50	4.09

Flexural rigidity *EI*

Sample	*Hosta x tardiana* 'El Niño' [Nmm^2]	*Caladium bicolor* [Nmm^2]	*Hemigraphis alternata* [Nmm^2]	*Pilea peperomioides* [Nmm^2]
1	9687.71	19876.04	199.99	368.68
2	10537.84	17379.01	218.55	695.19
3	3756.99	21060.04	140.39	448.71
4	13037.24	21595.13	347.42	733.84
5	5709.33	21126.63	221.57	484.31
6	12363.06	18563.03	193.30	596.39
7	14239.29	19070.92	166.88	376.10
8	7316.64	28818.30	345.73	746.83
9	8507.79	29424.98	257.69	528.00
10	6186.50	5441.03	192.98	387.58
11	6928.57	12909.90	293.34	451.45
12	6259.15	28566.33	163.89	791.90
13	6710.39	11519.63	156.85	498.80
14	12868.09	17253.69	305.11	1081.09
15	5245.05	17287.03	212.16	269.85
16	13493.57	14669.87	309.33	503.88
17	9456.00	12010.24	170.22	819.47
18	8938.77	30594.79	268.71	320.34
19	9093.48	18662.16	143.48	1063.68
20	8729.34	13896.60	210.98	1400.88
21	12066.06	15660.32	161.27	660.50
22	12181.08	15591.91	237.04	346.99
23	7608.80	17463.92	236.51	559.20
24	14895.90	14184.42	112.89	366.74
25	8065.56	22064.34	159.21	390.16
**Median**	8938.77	17463.92	210.98	503.88
**IQR**	5252.51	6456.76	93.79	346.27

Torsional rigidity *GK*

Sample	*Hosta x tardiana* 'El Niño'	*Caladium bicolor*	*Hemigraphis alternata*	*Pilea peperomioides*
	[Nmm^2]	[Nmm^2]	[Nmm^2]	[Nmm^2]
1	365.45	505.61	16.27	15.27
2	332.20	551.52	19.37	55.72
3	212.99	537.37	14.90	32.06
4	488.48	565.44	19.80	53.77
5	311.28	699.56	16.01	57.90
6	425.13	560.53	17.79	54.56
7	564.97	598.51	19.58	24.57
8	334.72	507.69	26.75	42.67
9	347.40	570.04	21.01	37.21
10	305.45	133.61	16.83	41.25
11	224.48	444.82	33.75	52.23
12	299.01	403.28	15.90	76.02
13	367.19	360.00	18.18	32.64
14	372.82	447.58	16.28	81.29
15	268.18	431.68	16.71	25.18
16	430.74	476.65	24.60	24.11
17	409.39	248.43	14.62	58.37
18	377.76	731.40	20.58	28.57
19	501.72	391.56	19.10	51.07
20	395.28	383.53	10.46	107.36
21	478.94	375.13	15.92	58.65
22	492.21	424.07	23.82	19.19
23	392.55	354.78	15.44	29.26
24	479.65	539.22	14.05	27.87
25	406.64	469.35	19.15	33.84
**Median**	377.76	469.35	17.79	41.25
**IQR**	98.54	159.96	3.88	27.16

Sample	***Hosta x tardiana* 'El Niño'**	***Caladium bicolor***	***Hemigraphis alternata***	***Pilea peperomioides***
	[-]	[-]	[-]	[-]
1	23.73	67.73	19.57	30.95
2	26.07	62.89	23.05	19.73
3	20.36	95.33	12.97	27.09
4	27.29	78.99	36.02	21.44
5	16.90	62.77	12.18	16.33
6	30.38	47.16	13.34	20.71
7	26.65	55.25	17.55	22.73
8	23.21	147.46	25.43	22.14
9	28.61	112.75	17.09	16.34
10	18.69	32.57	20.56	14.14
11	18.38	41.61	16.46	11.57
12	22.85	103.96	16.31	14.96
13	21.04	63.73	13.62	20.43
14	36.01	122.40	31.37	19.25
15	15.23	34.41	18.97	16.67
16	27.56	80.77	22.49	44.44
17	19.40	52.17	16.42	16.28
18	22.14	83.77	19.58	17.24
19	17.11	79.98	11.86	31.89
20	20.48	62.77	36.77	18.03
21	17.34	100.61	18.20	16.31
22	26.01	51.18	18.86	22.32
23	16.89	45.96	23.60	26.22
24	26.03	38.37	13.67	20.16
25	15.87	123.68	15.39	14.23
**Median**	22.14	63.73	18.20	19.73
**IQR**	7.69	44.15	7.10	5.99

Bending elastic modulus *E*

**Sample**	***Hosta x tardiana* 'El Niño'**	***Caladium bicolor***	***Hemigraphis alternata***	***Pilea peperomioides***
	[MPa]	[MPa]	[MPa]	[MPa]
1	149.65	189.68	164.53	84.57
2	142.39	171.61	198.39	81.91
3	74.92	241.85	107.38	142.10
4	213.83	243.59	225.09	119.29
5	110.09	183.65	229.58	123.93
6	213.96	144.41	110.97	85.09
7	151.42	139.50	201.32	152.47
8	148.12	405.42	161.80	122.68
9	132.88	448.07	158.44	107.40
10	111.94	40.60	193.33	106.04
11	111.90	140.35	139.31	98.47
12	110.76	269.80	104.84	85.54
13	66.16	197.77	106.92	106.87
14	229.27	279.05	280.16	126.11
15	115.56	122.09	171.26	76.71
16	212.61	241.09	245.72	89.11
17	154.98	126.96	136.08	143.91
18	128.38	311.80	229.53	106.50
19	113.14	192.19	172.65	113.03
20	121.56	182.01	285.56	140.37
21	145.70	312.92	116.41	142.86
22	215.16	196.88	194.88	96.95
23	85.62	98.73	175.79	165.33
24	276.75	148.45	109.64	115.73
25	132.29	306.72	167.77	110.25
**Median**	132.88	192.19	171.26	110.25
**IQR**	43.04	125.39	65.24	29.16

Torsional modulus *G*

**Sample**	***Hosta x tardiana* 'El Niño'**	***Caladium bicolor***	***Hemigraphis alternata***	***Pilea peperomioides***
	[MPa]	[MPa]	[MPa]	[MPa]
1	6.31	2.80	8.41	2.73
2	5.46	2.73	8.61	4.15
3	3.68	2.54	8.28	5.25
4	7.84	3.08	6.25	5.56
5	6.52	2.93	18.84	7.59
6	7.04	3.06	8.32	4.11
7	5.68	2.53	11.47	6.71
8	6.38	2.75	6.36	5.54
9	4.64	3.97	9.27	6.57
10	5.99	1.25	9.40	7.50
11	6.09	3.37	8.46	8.51
12	4.85	2.60	6.43	5.72
13	3.14	3.10	7.85	5.23
14	6.37	2.28	8.93	6.55
15	7.59	3.55	9.03	4.60
16	7.71	2.99	10.93	2.00
17	7.99	2.43	8.29	8.84
18	5.80	3.72	11.72	6.18
19	6.61	2.40	14.56	3.54
20	5.94	2.90	7.77	7.78
21	8.40	3.11	6.40	8.76
22	8.27	3.85	10.33	4.34
23	5.07	2.15	7.45	6.31
24	10.63	3.87	8.02	5.74
25	8.34	2.48	10.90	7.75
**Median**	6.37	2.90	8.46	5.74
**IQR**	2.03	0.59	2.49	2.90

Safety factors *SF*

**Sample**	***Hosta x tardiana* 'El Niño'**	***Caladium bicolor***	***Hemigraphis alternata***	***Pilea peperomioides***
	[-]	[-]	[-]	[-]
1	2.26	1.64	3.31	2.09
2	2.07	1.64	2.21	1.89
3	2.34	1.80	3.55	2.47
4	2.33	1.60	3.23	1.78
5	1.95	1.53	3.60	2.30
6	2.70	1.70	3.22	2.46
7	3.09	1.73	3.06	2.60
8	2.55	2.21	2.11	1.99
9	2.00	2.52	3.32	2.13
10	2.25	1.10	2.66	2.31
11	2.24	1.66	3.04	2.35
12	2.22	1.91	2.31	2.10
13	2.35	1.68	2.63	2.18
14	2.72	1.90	3.69	1.92
15	2.18	1.40	2.68	1.89
16	3.17	1.79	2.81	2.26
17	2.54	1.62	2.99	2.55
18	2.75	1.77	2.32	2.22
19	2.82	1.55	2.94	1.68
20	2.16	1.88	3.79	1.77
21	2.44	1.62	2.70	1.93
22	2.76	1.48	2.56	2.12
23	2.15	1.36	2.57	2.71
24	2.44	1.34	3.15	1.88
25	2.95	1.97	2.65	2.75
**Median**	2.35	1.66	2.94	2.13
**IQR**	0.50	0.25	0.59	0.43
