# Supplementary material for: Twist-to-Bend Ratios and Safety Factors of Petioles Having Various Geometries, Sizes and Shapes
Source: Front Plant Sci. 2021 Nov 11;12:765605. doi: 10.3389/fpls.2021.765605 (PMC8632552; doi:10.3389/fpls.2021.765605)
Supplement: Supplementary file 2 [file Data_Sheet_2.PDF]

These results represent the *p*-values calculated by comparing the different variables for the petioles of each studied species by post-hoc pairwise Wilcoxon tests in R.

**Aspect ratios *AR***	**unpaired pairwise.wilcoxon *p*-values**		*n* = 25
	Hosta x tardiana 'El Nino'	*Caladium bicolor*	*Hemigraphis alternata*
Caladium bicolor	0.0000	-	-
Hemigraphis alternata	0.0336	0.0000	-
Pilea peperomioides	0.0000	0.0001	0.0000

***I/J* ratios**	**unpaired pairwise.wilcoxon *p*-values**		*n* = 25
	Hosta x tardiana 'El Nino'	*Caladium bicolor*	*Hemigraphis alternata*
Caladium bicolor	0.0000	-	-
Hemigraphis alternata	0.0000	0.0000	-
Pilea peperomioides	0.0000	0.0000	0.0000

***I/K* ratios**	**unpaired pairwise.wilcoxon *p*-values**		*n* = 25
	Hosta x tardiana 'El Nino'	*Caladium bicolor*	*Hemigraphis alternata*
Caladium bicolor	0.0000	-	-
Hemigraphis alternata	0.0000	0.2630	-
Pilea peperomioides	0.0000	0.0340	0.0287

**Tapering modes *α***	**unpaired pairwise.wilcoxon *p*-values**		*n* = 25
	Hosta x tardiana 'El Nino'	*Caladium bicolor*	*Hemigraphis alternata*
Caladium bicolor	0.0000	-	-
Hemigraphis alternata	0.2976	0.0000	-
Pilea peperomioides	0.0145	0.0006	0.1908

**Lamina-petiole weight ratios *WR***	**unpaired pairwise.wilcoxon *p*-values**		*n* = 25
	Hosta x tardiana 'El Nino'	*Caladium bicolor*	*Hemigraphis alternata*
Caladium bicolor	0.0000	-	-
Hemigraphis alternata	0.0000	0.0000	-
Pilea peperomioides	0.0000	0.0000	0.0000

**Axial second moments of area *I***	**unpaired pairwise.wilcoxon *p*-values**		*n* = 25
	Hosta x tardiana 'El Nino'	*Caladium bicolor*	*Hemigraphis alternata*
Caladium bicolor	0.0121	-	-
Hemigraphis alternata	0.0000	0.0000	-
Pilea peperomioides	0.0000	0.0000	0.0000

**Polar second moments of area *J***	**unpaired pairwise.wilcoxon *p*-values**		*n* = 25
	Hosta x tardiana 'El Nino'	*Caladium bicolor*	*Hemigraphis alternata*
Caladium bicolor	0.0000	-	-
Hemigraphis alternata	0.0000	0.0000	-
Pilea peperomioides	0.0000	0.0000	0.0000

**Torsion constants *K***	**unpaired pairwise.wilcoxon *p*-values**		*n* = 25
	Hosta x tardiana 'El Nino'	*Caladium bicolor*	*Hemigraphis alternata*
Caladium bicolor	0.0000	-	-
Hemigraphis alternata	0.0000	0.0000	-
Pilea peperomioides	0.0000	0.0000	0.0000

**Twist-to-bend ratios *EI/GK***	**unpaired pairwise.wilcoxon *p*-values**		*n* = 25
	Hosta x tardiana 'El Nino'	*Caladium bicolor*	*Hemigraphis alternata*
Caladium bicolor	0.0000	-	-
Hemigraphis alternata	0.0000	0.0000	-
Pilea peperomioides	0.0000	0.0000	0.0916

**Flexural rigidities *EI***	**unpaired pairwise.wilcoxon *p*-values**		*n* = 25
	Hosta x tardiana 'El Nino'	*Caladium bicolor*	*Hemigraphis alternata*
Caladium bicolor	0.0000	-	-
Hemigraphis alternata	0.0000	0.0000	-
Pilea peperomioides	0.0000	0.0000	0.0000

**Torsional rigidities *GK***	**unpaired pairwise.wilcoxon p-values**		
	Hosta x tardiana 'El Nino'	*Caladium bicolor*	*Hemigraphis alternata*
Caladium bicolor	0.0055	-	-
Hemigraphis alternata	0.0000	0.0000	-
Pilea peperomioides	0.0000	0.0000	0.0000

***E/G* ratios**	**unpaired pairwise.wilcoxon *p*-values**		*n* = 25
	Hosta x tardiana 'El Nino'	*Caladium bicolor*	*Hemigraphis alternata*
Caladium bicolor	0.0000	-	-
Hemigraphis alternata	0.2128	0.0000	-
Pilea peperomioides	0.2243	0.0000	0.6305

**Elastic moduli *E***	**unpaired pairwise.wilcoxon *p*-values**		*n* = 25
	Hosta x tardiana 'El Nino'	*Caladium bicolor*	*Hemigraphis alternata*
Caladium bicolor	0.0170	-	-
Hemigraphis alternata	0.1084	0.2092	-
Pilea peperomioides	0.0305	0.0000	0.0000

**Torsional moduli *G***	**unpaired pairwise.wilcoxon *p*-values**	*n* = 25	
	Hosta x tardiana 'El Nino'	*Caladium bicolor*	*Hemigraphis alternata*
Caladium bicolor	0.0000	-	-
Hemigraphis alternata	0.0000	0.0000	-
Pilea peperomioides	0.3067	0.0000	0.0000

**Safety factors *SF***	**unpaired pairwise.wilcoxon *p*-values**	*n* = 25	
	Hosta x tardiana 'El Nino'	*Caladium bicolor*	*Hemigraphis alternata*
Caladium bicolor	0.0000	-	-
Hemigraphis alternata	0.0011	0.0000	-
Pilea peperomioides	0.0048	0.0000	0.0000

Colour code

p >= 0.05 not significant

p < 0.05 significant
